# Climate and soil stressed elevation patterns of plant species to determine the aboveground biomass distributions in a valley-type Savanna

**DOI:** 10.3389/fpls.2024.1324841

**Published:** 2024-03-27

**Authors:** Guangxiong He, Zhengtao Shi, Haidong Fang, Liangtao Shi, Yandan Wang, Haozhou Yang, Bangguo Yan, Chaolei Yang, Jianlin Yu, Qiaoling Liang, Lei Zhao, Qin Jiang

**Affiliations:** ^1^ Yunnan Key Laboratory of Plateau Geographical Processes and Environmental Change, Faculty of Geography, Yunnan Normal University, Kunming, Yunnan, China; ^2^ Tropical Eco-Agriculture Research Institute, Yunnan Academy of Agricultural Sciences, Yuanmou, Yunnan, China; ^3^ Yuanmou Dry-hot Valley Botanical Garden, Yunnan Academy of Agricultural Sciences, Yuanmou, Yunnan, China; ^4^ National Soil and Water Conservation Science and Technology Demonstration Park of Yunnan Yuanmou Jinlei, Yunnan Academy of Agricultural Sciences, Yuanmou, Yunnan, China; ^5^ Kunming General Survey of Natural Resources Center, China Geological Survey, Kunming, Yunnan, China

**Keywords:** species diversity, aboveground biomass, valley-type Savanna, RDA, environmental variables and factors

## Abstract

**Introduction:**

Extreme environments such as prolonged high temperatures and droughts can cause vulnerability of vegetation ecosystems. The dry-hot valleys of Southwestern China, known for their extremely high annual temperature, lack of water, and unique non-zonal “hot island” habitat in the global temperate zone, provide exceptional sites for studying how plant adapts to the prolonged dry and hot environment. However, the specific local biotic-environment relationships in these regions remain incompletely elucidated. The study aims to evaluate how valley-type Savanna vegetation species and their communities adapt to long-term drought and high-temperature stress environments.

**Methods:**

The study investigated the changes in species diversity and communities’ aboveground biomass of a valley-type Savanna vegetation along an elevation gradient of Yuanmou dry-hot valley in Jinsha River basin, southwest China. Subsequently, a general linear model was utilized to simulate the distribution pattern of species diversities and their constituent biomass along the elevation gradient. Finally, the RDA and VPH mothed were used to evaluate the impacts and contributions of environmental factors or variables on the patterns.

**Results and discussion:**

The field survey reveals an altitudinal gradient effect on the valley-type Savanna, with a dominant species of shrubs and herbs plants distribution below an elevation of 1700m, and a significant positive relationship between the *SR*, *Shannon-Wiener*, *Simpson*, and *Pielou* indices and altitudes. Relatively, the community aboveground biomass did not increase significantly with elevation, which was mainly due to a decreased biomass of herbaceous plants along the elevation. Different regulators of shrub-herbaceous plant species and their functional groups made different elevation patterns of species diversity and aboveground biomass in valley-type Savannas. Herbaceous plants are responsible for maintaining species diversity and ensuring stability in the aboveground biomass of the vegetation. However, the influence of shrubs on aboveground biomass became more pronounced as environmental conditions varied along the altitudinal gradient. Furthermore, species diversity was mainly influenced by soil and climatic environmental factors, whereas community biomass was mainly regulated by plant species or functional groups. The study demonstrates that the spatial pattern of valley-type Savanna was formed as a result of different environmental responses and the productive capacity of retained plant species or functional groups to climate-soil factors, highlighting the value of the Yuanmou dry-hot Valley as a microcosm for exploring the intricate interactions between vegetation evolution and changes in environmental factors.

## 1 Introduction

Species diversity reflects the underlying structure and functioning of ecosystems, while aboveground biomass serves as an indicator of vegetation traits and productivity. The intrinsic interplay between biodiversity and biomass intricately shapes the functionality and stability of vegetation ecosystems (Tilman et al., 1996; Tilman, 2000; Loreau et al., 2001; Bai et al., 2004). The complex interrelations between diversified topography and rapidly shifting microclimates inherent to mountainous systems (Körner, 2000), render them responsive and sensitive to novel factors like human activities and climate change (Rangwala and Miller, 2012; Liang et al., 2020; Wang et al., 2021). These systems directly or indirectly manifest the vertical and horizontal zonality of mountain landscapes (Richards et al., 1975; Körner, 2000; Kou et al., 2020), and significantly contribute to the advancement of essential understandings such as global-scale vegetation distribution patterns (Rahbek et al., 2019). Altitude, a comprehensive condensed indicator reflecting mountainous ecological environments, induces changes in environmental variables including temperature, humidity, and solar radiation, thereby influencing plant biomass and diversity distribution patterns (Gaston, 2000; Wang et al., 2021). Therefore, a comprehensive exploration of the altitudinal patterns of plant species diversity and biomass in mountainous environments holds profound significance for the conservation of ecosystem biodiversity, the maintenance of productivity, and ecological restoration.

In mountainous environments, various factors such as climate, space, and soil exhibit spatiotemporal differentiation along elevation gradients, leading to five main distribution patterns of elevation gradients in the diversity of plant species or community productivity (He et al., 1997, Liu et al., 2005; Liu, 2021): including linear negative correlations, linear positive correlations, higher values at moderate elevations, lower values at moderate elevations, and no linear correlation. The main influencing mechanisms include two major perspectives of environmental or biological determinism and community species coexistence mechanisms (Liang et al., 2022), along with seven possible pathways of action (Rahbek et al., 2019), involving various mechanisms such as spatial heterogeneity theory, niche theory, and neutral effects (Niu et al., 2009; Liang et al., 2022; Niu et al., 2022), and the development of modern coexistence theory (Wang et al., 2022). However, there is still a lack of unified understanding of the response mechanisms of species diversity and community productivity along elevation gradients in mountainous environments, which remains a hot research topic in biogeography, ecology, and other related fields. Additionally, because species diversity and community productivity do not always exhibit a consistent and explanatory relationship, there exist four types of related relationships: promoting effects, inhibiting effects, unimodal curves, and unrelated relationships (Guo et al., 2019). The elevation gradient patterns of species diversity or community productivity along different climate zones or latitudinal gradients also show significant differences (Hodkinson, 2005, Cuesta et al., 2017). Therefore, the relationships between species diversity and community productivity along elevation gradients, as well as their mechanisms, require further in-depth research.

The mountainous region of Himalayan-Hengduan stands as a global biodiversity hotspot, boasting a complex and dynamic landscape. The lower sections of rivers like Jinsha, Lancang, Nu, and Yuan exhibit undulating terrains with steep slopes, deep valleys, and altitudinal variations spanning 200-1000m. This unique geographical context engenders distinctive arid valley climates, facilitating the coexistence of diverse biological traits (Zhang, 1992; Zheng et al., 2000). In this context, the arid valley of the Jinsha River assumes a prominent role. It stands out as a “dry zone” developed within the backdrop of the global temperate humid climate (Zhang, 1992), characterized by a high aridity index of 21.2 during the dry season (Liu et al., 2011). Geobotanically, this region reveals the unusual landscape of tropical sparse grassland ecosystems interspersed within temperate evergreen broad-leaved forests, encapsulating remnants of valley-type savanna vegetation strikingly reminiscent of tropical counterparts (Shen, 2016). The species composition exhibits a pan-tropical and subtropical typology (Jin et al., 1987). Geomorphologically, the arid valley arises as a “lowland” incised within ancient planation surfaces (Zhang, 1992). These features bestow the limited projection area with a broad range of altitudinal gradients, hydrothermal environments, and dramatic vegetational transformations, rendering it an exceptional laboratory for investigating biological-environmental relationships.

The unique savanna vegetation within the arid valley of the Jinsha River has garnered considerable attention. Initial studies predominantly focused on the zonality of vegetation landscape appearance (Qu, 1957; Jiang, 1980), taxonomic compositions (Wu, 1965, Wu, 1979), and community classifications (Jin et al., 1987; Ou, 1988). Driven by the requirements of regional vegetation restoration and soil degradation mitigation, subsequent efforts also delved into the functional traits underpinning the adaptive evolution of arid valley vegetation (Zhang et al., 2012; Yan et al., 2016) and the physiochemical intricacies (Yang et al., 2023). During this period, vertical differentiation of vegetation arising from complex interactions between topography and regional climate emerged as a focal ecological concern. Along the latitudinal gradient, the productivity of arid valley communities exhibits a monotonous increase with latitude (Wang et al., 2022). In vertical gradients, the Three Parallel Rivers region showcases an elevation-dependent increase in herbaceous and shrub species richness (Yang et al., 2016), reflecting patterns of community and floristic spatial differentiation correlated with environmental gradients (Liu et al., 2016). These investigations contribute invaluable insights into the evolutionary adaptation and ecological functional shifts of arid valley vegetation. However, due to the complexity and ecological sensitivity of mountain-valley environments (Hua et al., 2021), coupled with the intensified impacts of human activities and climate change on fragile mountain ecosystems (Beniston, 2003; Albrich et al., 2020), larger-scale studies fail to precisely capture the intricate relationships between local-scale biology and environment (Feldmeier et al., 2020). Thus, a focused investigation into local-scale species diversity within the arid valley, coupled with the elucidation of environmental factors driving vertical distribution patterns, remains imperative.

The Yuanmou dry-hot valley stands as an emblematic archetype within the panorama of arid valleys in southwestern China. It is emblematic of regions characterized by paucity of water, prolonged hot, and dispersion of valley-type savanna vegetation, constitutes the focal arena of inquiry (Jin et al., 1987; He et al., 2018). To enhance our theoretical understanding of spatial heterogeneity within vegetation and the mechanisms shaping the formation and evolution of vegetation distribution patterns in the distinctive habitat of dry-hot valleys, this study focuses on the Yuanmou County segment of the Jinsha River, southwest China. Through field-based vegetation surveys, we comprehensively examine the impact of regional topographical features, hydro-energy environmental factors, and soil characteristics on pattern formation. The study addresses two pivotal scientific inquiries: (1) How do species diversity and biomass of Valley-type Savanna communities vary across different altitudes? (2) What are the primary drivers and underlying mechanisms behind alterations in species diversity and biomass along the altitudinal gradient in Valley-type Savanna communities? The findings of this research would offer a foundational framework for devising strategies in the preservation and restoration management of vulnerable mountainous ecosystems of dry-hot valleys.

## 2 Materials and methods

### 2.1 Study site

**Figure 1:**
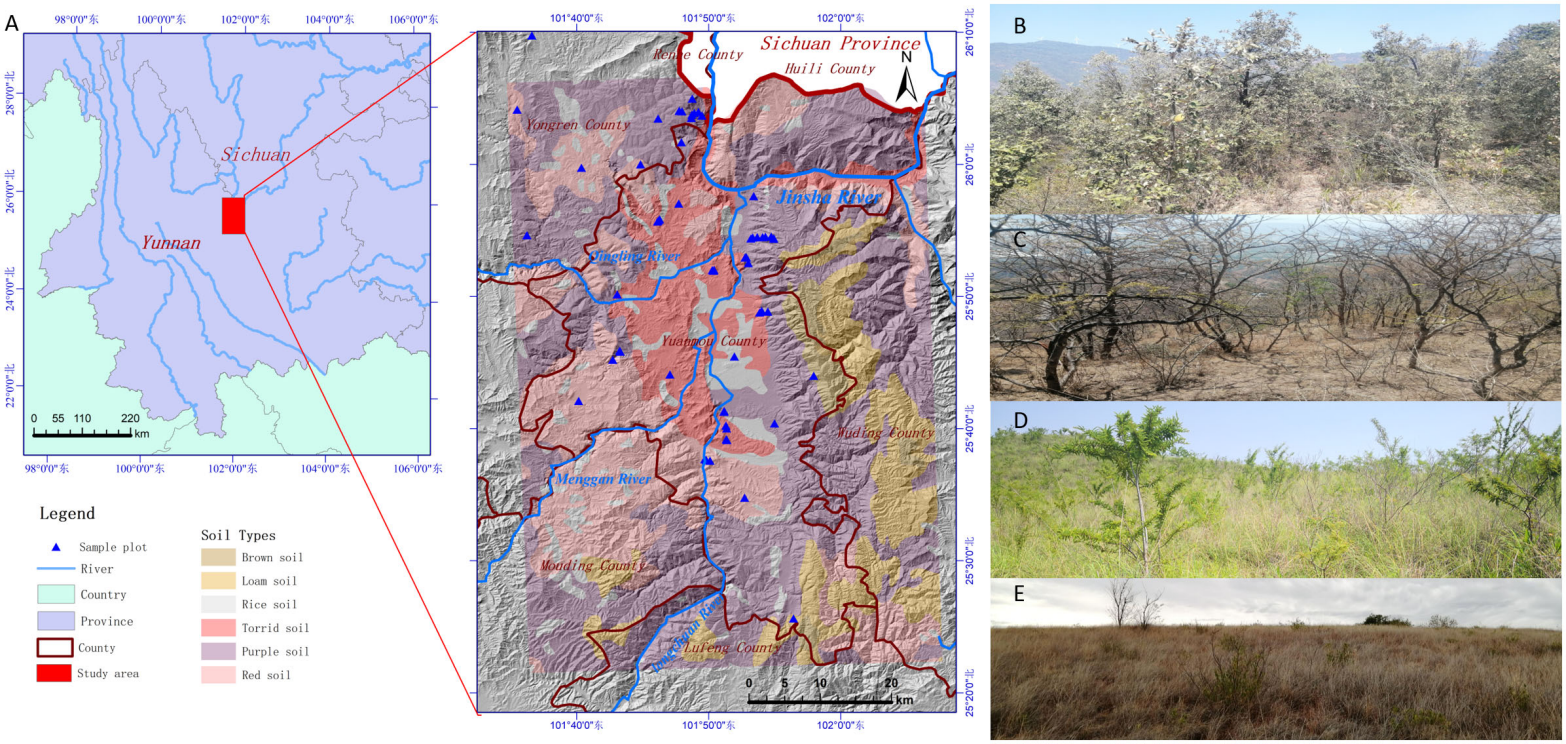
Study area. **(A)** Location, mountains, soil types, and the sample plot. **(B)** Temperate forest. **(C)** Savanna shrub **(D)** Savanna shrub and grassland, and **(E)** Savanna grassland.

The investigation was carried out in the dry-hot valley of Yuanmou County (**Figure 1A**), a section of the Jinsha River basin in Yunnan Province, southwest China (between latitude 25°23’–26°06’N and longitude 101°35’–102°06’E, with elevations ranging from 898–2823 meters). High mountains and deep valleys are a distinctive feature of Yuanmou County, with the primary tributary of the Longchuan River joining the Jinsha River to form a river-lake basin known as the dry-hot valley of Yuanmou basin (Zhang et al., 2000). The regional vegetation of valley-type Savanna, which is primarily made up of *Lannea coromandelica*, *Polyalthia cerasoides*, *Campylotropis delavayi*, and *Heteropogon contortus* (He et al., 2018, He et al., 2023), is found below 1600 meters in elevation, where experiences a dry-hot monsoon climate similar to that of southern Asia (Jin et al., 1987; Ou, 1988). Red soil, purple soil, and torrid red soil are the three basic soil types (He and Huang, 1995). The annual potential evaporation is 3847.8 mm, and the annual average precipitation is 634.0 mm. Between May and October, 90.5% of the total precipitation falls, while the dry season, which lasts from November to April of the following year, has a dryness index of 21.2 (Liu et al., 2011). The average annual sunshine is 2670.4 hours, with an annual average temperature of 21 °C, a maximum temperature of 42 °C, a minimum temperature of -0.8 °C, and an annual accumulated temperature of 7996 °C for temperatures ≥ 10 °C (He et al., 2018).

### 2.2 Vegetation investigation

The typical community inventory was conducted using the plot-quadrat survey method (Fang et al., 2009). The tree-shrub-grassland community component and aboveground biomass were surveyed in August-September 2022, when the vegetation structure was the most complete, within the altitude range of 1000-2000 meters in Yuanmou dry-hot Valley. Sample plots were sated in different altitudes without obvious artificial disturbances. Three quadrats were nested in each sample plot according to the tree layer (10 m x 10 m), shrub layer (5 m x 5 m), and herb layer (1 m x 1 m), and the species name, number of individuals, average height, and average crown diameter of the species in each quadrat were recorded. This study obtained data from 111 community plots and 97 species.

The aboveground biomass of communities and species was determined using the harvest method. This study mainly focused on the characteristics of plant diversity and biomass changes along the altitude gradient of the valley-type Savanna vegetation. To reduce damage to the fragile vegetation ecosystem in the study area, only one shrub layer (5 m × 5 m) and one herb layer (1 m × 1 m) quadrat were harvested from each community plot. Different species were separately harvested and weighed to obtain fresh biomass and dry biomass dried at 65°C to a constant weight in the laboratory. This study obtained data from 38 shrub sample plots and 111 herb sample plots. The fresh and dry biomass of different species in the shrub and herb layers were converted to g/m^2^, and the species importance value (*IV_i_*) was calculated using Equation 1 (He et al., 2023).


(1) $$IV_{i}=\frac{Biomass_{species}}{Biomass_{plot}}$$

Additionally, to quantify the components of community aboveground biomass, the vegetation species were divided into 3 plant functional groups according to the clear landscape changes of trees, shrubs, and grasses lifeforms (Jobbágy et al., 1996).

### 2.3 Soil sample selection and characteristics determination

Soil sample collection and vegetation sample survey are conducted simultaneously. Soil samples were drilled and collected from the 0-20 cm soil layer along the diagonal of each forest plot mentioned above. A total of 5 drills were taken from each plot and mixed into one soil sample. Select gravel and roots from soil samples, drying them in a cool and dry place and through a 2mm sieve for constant nutrient determination. The content of total carbon (TC), total nitrogen (TN), hydrogen (Element_H), and sulfur (Element_S) in soil was measured using an elemental analyzer (Vario MACRO cube, Elemental, Langenselbold, Germany).

### 2.4 Community diversity index

Biodiversity is an important characteristic of a biological community, reflecting its characteristics and the relationship between the community and the environment. There are many indicators indicating community diversity, such as α diversity is the number of species in the community, namely species richness (including number of species, S, and Shannon-Wiener index, H, Equations 2, 3), and the relative density of species in the community, namely species evenness (including Simpson index, D, and Pielou index, E, Equations 4, 5) (Fang et al., 2009). The following indicators measured the valley-type Savanna community diversity and they were computed using a vegan package (Oksanen et al., 2022):


(2) S=N

Shannon-Wiener index:


(3) $$H=-\sum_{i=1}^{S}p_{i}lnp_{i}$$

Simpson index:


(4) $$D=1-\sum_{i=1}^{S}p_{i}^{2}$$

Pielou index:


(5) $$E=\frac{1-D}{1-\frac{1}{S}}$$

Where *N* is the number of species, and pi is the important value.

### 2.5 Climate and environment data

Bioclimatic variables mainly include energy (including temperature) and moisture (including precipitation). Annual trends (such as mean annual temperature and annual precipitation), seasonality (such as range of temperature and precipitation throughout the year), and extreme or limiting environmental factors (such as temperature in the coldest and hottest months, and precipitation of moisture and precipitation) were used to represent the environmental characteristics of a region (Fang et al., 2002; Hawkins et al., 2003). WorldClim (https://worldclim.org/maps/index.html) is a database of high spatial resolution global weather and climate data used for mapping and spatial modeling and 19 bioclimatic variables covering the world, including annual mean temperature and annual mean precipitation, were calculated by a resolution of 1 km (Fick and Hijmans, 2017). This study adopts the approach developed by Liu (Liu, 2015) to calculate the bioclimatic indicators: Initially, we gathered long-term observational data from 20 ground-based meteorological stations within the study area (20 stations’ rainfall and temperature per day data around the study area were downloaded from the Resource and Environmental Science Data Center of the Chinese Academy of Sciences (http://www.resdc.cn), 1990-2020, 30 years), and 19 bioclimatic indices for these stations were calculated as WorldClim definitions, serving as authentic references. Subsequently, corresponding data from the WorldClim dataset for the same 19 indices were extracted from 20 meteorological stations surrounding the study area. These data constitute simulated values for analysis. Then, a robust regression analysis links the actual and simulated data to yield residual correction maps that play a vital role in the subsequent adjustments. In the final step, these correction maps were utilized to recalibrate a larger portion of the WorldClim dataset, encompassing an area three times greater than the primary study region. Concurrently, field survey data provided geospatial coordinates for sample points, allowing us to gather comprehensive information on the 19 bioclimatic indices. This methodology was seamlessly integrated within ArcGIS 10.8 software.

### 2.6 Data analysis

Using the plots as the statistical unit, the community biodiversity index and component aboveground biomass were calculated, and a general linear model was used to simulate the pattern of community biodiversity and its component biomass along the altitude gradient at two levels of environmental variables and factors. The redundancy analysis (*RDA*) was used to analyze the effects of environmental factors or variables on plant species diversity and aboveground biomass. During the process, the 4 factors and their 36 environmental variables (see **Table 1**) were set as explanatory variables, and community biodiversity indexes of SR, Shannon-Wiener, Simpson, and Pielou as response variables for plant species diversity pattern analysis, and community aboveground biomass of fresh, dry, and litters as response variables for aboveground biomass pattern analysis. Then, hierarchical partitioning (*HP*) was used to assign separate effects to each explanatory variable and assess the relative importance of individual environmental variables. To quantify and test the explanatory power of individual variables and combined environmental factors on the pattern of community species diversity and component biomass along altitude gradient, variables were classified into four categories: space, biotic, soil, and climate factors. The variance inflation factor (*VIF*) was used to remove the collinearity of environmental variables with values less than 10, and the component variables of the four categories of environmental factors are determined separately. Variation partitioning analysis (*VPA*) was then used to calculate the contribution rates of environmental factors and their combinations. Statistical analysis and plotting were performed using the *vegan* package (Oksanen et al., 2022), *rdacca.hp* package (Lai et al., 2022; Liu et al., 2023), and the *ggplot2* package (Wickham, 2016) in *R* software version 4.2.1 (R Core Team, 2022).

**Table 1:** 36 environmental variables of Yuanmou dry-hot valley in Jinsha River basin, southwest China (n=111).

	Variables	max	min	Mean ± SE
Space factors	Latitude	25.93	25.54	25.79 ± 0.00
	Longitude	101.94	101.71	101.85 ± 0.00
	Elevation	2027.95	998.55	1345.81 ± 2.32
	Slope	45.00	1.00	17.95 ± 0.08
	Aspect	353.00	6.00	198.15 ± 0.81
	ym19_Curva	4.49	0.64	0.08 ± 0.01
Vegetation factors	Cover	95.00	30.00	70.79 ± 0.14
	Individual	348.00	6.00	67.57 ± 0.47
	Species richness	19.00	2.00	6.81 ± 0.03
	Shannon.Wiener	2.55	0.15	1.25 ± 0.01
	Simpson	0.90	0.07	0.58 ± 0.00
	InSimpson	10.18	1.07	3.24 ± 0.02
	Pielou	0.98	0.21	0.68 ± 0.00
Climate factors (energy)	BIO1 = Annual Mean Temperature(°C)	22.13	16.73	20.34 ± 0.01
	BIO2 = Mean Diurnal Range(°C)	12.76	11.40	12.25 ± 0.00
	BIO3 = Isothermality (BIO2/BIO7×100)	49.26	47.70	48.74 ± 0.00
	BIO4 = Temperature Seasonality	458.99	439.04	451.65 ± 0.04
	BIO5 = Max Temperature of Warmest Month(°C)	33.00	26.70	30.97 ± 0.01
	BIO6 = Min Temperature of Coldest Month(°C)	7.60	2.80	5.83 ± 0.01
	BIO7 = Temperature Annual Range(°C)	25.90	23.90	25.14 ± 0.00
	BIO8 = Mean Temperature of Wettest Quarter(°C)	26.67	21.32	24.87 ± 0.01
	BIO9 = Mean Temperature of Driest Quarter(°C)	18.22	10.72	15.10 ± 0.02
	BIO10 = Mean Temperature of Warmest Quarter(°C)	26.85	21.33	25.00 ± 0.01
	BIO11 = Mean Temperature of Coldest Quarter(°C)	16.05	10.72	14.30 ± 0.01
Climate factors (Water)	BIO12 = Annual Precipitation (mm)	759.00	647.00	681.38 ± 0.26
	BIO13 = Precipitation of Wettest Month (mm)	163.00	143.00	148.65 ± 0.05
	BIO14 = Precipitation of Driest Month (mm)	8.00	4.00	5.12 ± 0.01
	BIO15 = Precipitation Seasonality (mm)	96.47	87.39	94.30 ± 0.02
	BIO16 = Precipitation of Wettest Quarter (mm)	438.00	385.00	401.91 ± 0.12
	BIO17 = Precipitation of Driest Quarter (mm)	28.00	14.00	18.22 ± 0.03
	BIO18 = Precipitation of Warmest Quarter (mm)	350.00	302.00	318.21 ± 0.10
	BIO19 = Precipitation of Coldest Quarter (mm)	28.00	15.00	18.46 ± 0.03
Soil factors	TN	0.15	0.01	0.05 ± 0.00
	TOC	3.48	0.25	0.95 ± 0.01
	Element_H	1.63	0.26	0.86 ± 0.00
	Element_S	77.94	0.39	14.30 ± 0.15

## 3 Result

### 3.1 Plant species diversity of valley-type Savanna

#### 3.1.1 Vegetation distribution pattern in Yuanmou

**Figure 2:**
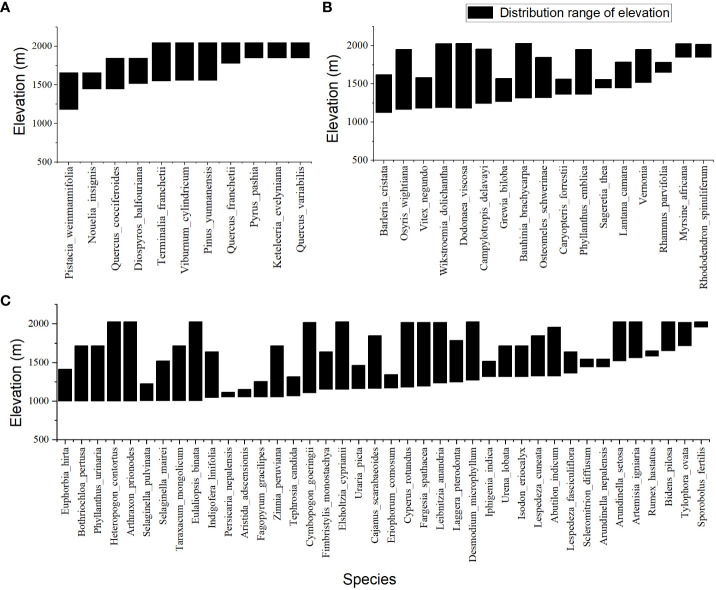
Plant species distribution pattern along an elevation gradient of Valley-type Savanna in Yuanmou hot dry valley. **(A)** Tree species. **(B)** Shrub species, and **(C)** Grass species.

Four vegetation types of temperate forest (**Figure 1B**), savanna shrub (**Figure 1C**), savanna shrub and grass (**Figure 1D**), and savanna grassland were identified in the study area. The species distribution below 2200 meters had a typical altitudinal gradient effect (**Figure 2**) and could be divided into three typical sections. In the section below 1400 meters, the dominant plants are herbaceous, with only a few trees or shrubs sparsely distributed in small patches along the river, and against the typical large grassland background, forming a “sparse tree grassland landscape” (**Figure 1E**). Typical species here include *Heteropogon contortus*, *Cymbopogon goeringii*, *Bothriochloa pertusa*, and occasional large-shaped individual tree species such as *Pistacia weinmannifolia*, *Nouelia insignis*, *Tamarindus indica*, or Bombax ceiba (**Figure 2**). In the section between 1400 meters and 1700 meters, shrubs showed an increasing importance of status and role in the community along the altitude, gradually replacing the dominant herbaceous plants at an altitude of 1400 meters, becoming the new dominant group, making a community of usually lacking trees and the vegetation-type transiting to a “shrub-grass landscape” (**Figure 1D**). Relatively exceptionally, a dry zone with a predominance of shrubs and extreme degradation of herbaceous plants occurs from 1600 to 1700 meters in the upper of the section, forming a “Savanna shrub landscape” (**Figure 1C**). Typical species here include shrubs of *Vitex negundo*, *Phyllanthus emblica*, *Dodonaea viscosa*, *Osteomeles schwerinae*, and occasional distribution of tree species such as *Terminalia franchetii* and *Diospyros balfouriana* (**Figure 2**). In the region above 1700 meters, tree species begin to appear and gradually replace the dominant position of shrubs at the altitude of 1800 meters, thus becoming the dominant group. The hierarchical vegetation structure of trees-shrubs-grasses tends to be perfect, and the “subtropical needle-leaf and broadleaf mixed forest-evergreen broadleaf forest landscape”(**Figure 1B**) appears in succession. Typical tree species here include *Terminalia franchetii*, *Pinus yunnanensis*, *Quercus cocciferoides*, *Quercus franchetii*, etc.

#### 3.1.2 Plant species diversity pattern of valley-type Savanna in Yuanmou

**Figure 3:**
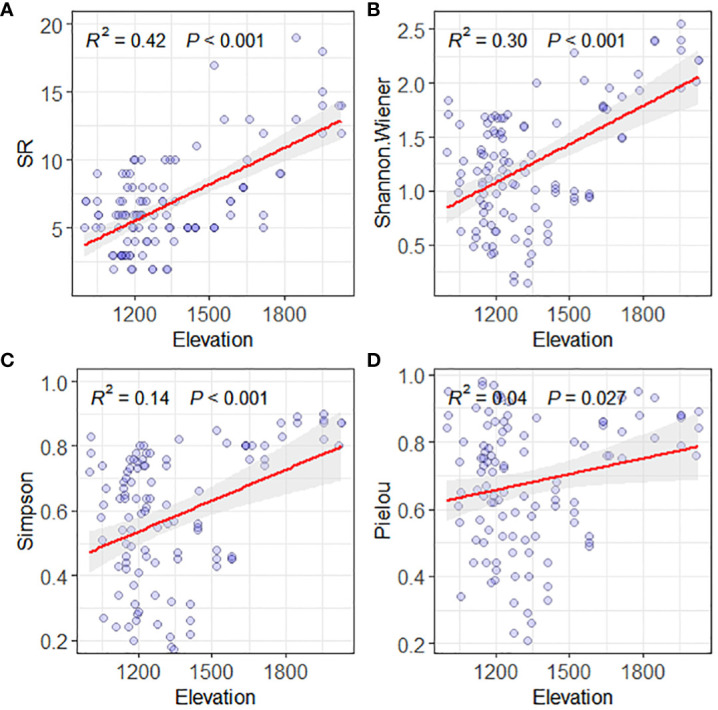
Plant species diversity pattern along an elevation gradient of Valley-type Savanna in Yuanmou hot dry valley. **(A)** SR for species richness, **(B)** Shannon Wiener index, **(C)** Simpson index, and **(D)** Pielou index.

The above result shows that the Savanna vegetation in the valley of the studied area is mainly distributed below an altitude of 1700 meters, with shrubs and herbaceous plants being the dominant group classifications. To include all the valley Savanna vegetation in the dry hot valley of Yuanmou, the altitude range of the study was extended to 2200 meters, and the altitudinal gradient pattern of species diversity in the “shrub-grass zone” community was analyzed. The results showed that the species richness indexes of SR and Shannon-Wiener, and species evenness indexes of Simpson and Pielou in the area all showed a significant increasing trend (*p* < 0.05) with altitude (**Figure 3**). This indicates that altitude has a significant positive impact (*p* < 0.05) on the distribution pattern of species diversity of the valley Savanna vegetation in the Yuanmou dry-hot valley zone by promoting species richness and evenness.

### 3.2 Aboveground biomass distribution pattern of valley-type Savanna

#### 3.2.1 Aboveground biomass distribution pattern in Yuanmou

The community’s above biomass components of valley-type Savanna vegetation were fresh biomass, dry biomass, and litter biomass below an altitude of 1700 m (**Table 2**). Specifically, at the community level, the mean fresh biomass of the vegetation was recorded as 396.23 ± 1.91 g/m², accompanied by an average dry biomass of 88.40 ± 0.40 g/m² and a litter biomass of 84.77 ± 0.38 g/m². Upon closer scrutiny of the shrub-herb component level, herbaceous plants exhibit an average fresh biomass of 367.99 ± 1.92 g/m², complemented by an average dry biomass of 156.63 ± 0.80 g/m² and a litter biomass of 106.41 ± 0.54 g/m². In contrast, shrubs demonstrate an average fresh biomass of 28.24 ± 0.59 g/m², accompanied by an average dry biomass of 13.34 ± 0.29 g/m² and a litter biomass of 17.08 ± 0.43 g/m². Progressing to the functional group level, all species collectively present an average fresh biomass of 72.25 ± 0.44 g/m², paired with an average dry biomass of 32.47 ± 0.19 g/m². Specifically, shrub species manifest an average fresh biomass of 39.03 ± 0.49 g/m², in tandem with an average dry biomass of 18.50 ± 0.25 g/m². In contrast, herbaceous species showcase an average fresh biomass of 73.51 ± 0.47 g/m², coupled with an average dry biomass of 32.98 ± 0.20 g/m².

**Table 2:** The aboveground biomass of valley-type savanna in Yuanmou hot dry valley (n=111).

Variables	max	min	Mean ± SE
Plot fresh biomass(g/m^2^)	1285.16	51.61	396.23 ± 1.91
Plot dry biomass(g/m^2^)	578.60	22.98	88.40 ± 0.40
Plot litter biomass(g/m^2^)	652.44	3.21	84.77 ± 0.38
Grass fresh biomass(g/m^2^)	1283.54	50.69	367.99 ± 1.92
Grass dry biomass(g/m^2^)	577.95	12.08	156.63 ± 0.80
Grass litter biomass(g/m^2^)	331.59	3.01	106.41 ± 0.54
Shrub fresh biomass(g/m^2^)	512.45	0.62	28.24 ± 0.59
Shrub dry biomass(g/m^2^)	254.62	0.25	13.34 ± 0.29
Shrub litter biomass(g/m^2^)	366.26	0.19	17.08 ± 0.43
Fresh biomass per species(g·m^2^)	263.09	13.06	72.25 ± 0.44
Dry biomass per species(g·m^2^)	104.62	2.90	32.47 ± 0.19
Fresh biomass per shrub(g·m^2^)	256.23	1.08	39.03 ± 0.49
Dry biomass per shrub(g·m^2^)	127.31	0.38	18.50 ± 0.25
Fresh biomass per grass(g·m^2^)	263.09	12.64	73.51 ± 0.47
Dry biomass per grass(g·m^2^)	117.96	1.52	32.98 ± 0.20

#### 3.2.2 Aboveground biomass distribution of the community components in Yuanmou

**Figure 4:**
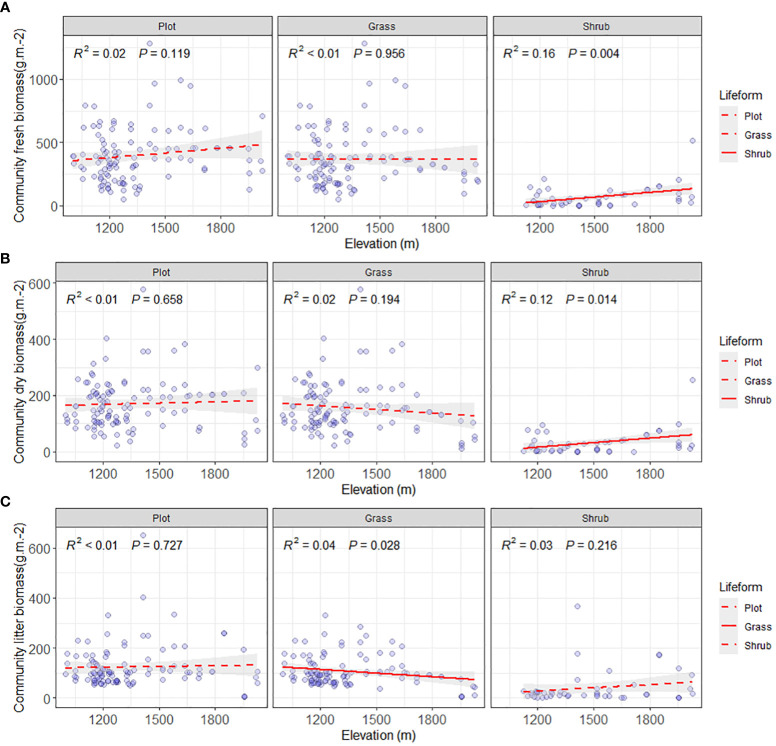
Comparison of aboveground biomass on community level. **(A)** Aboveground community fresh biomass on plot, grass and shrub level (g/m^2^), **(B)** Aboveground community dry biomass on plot, grass and shrub level (g/m^2^), and **(C)** aboveground community litter biomass on plot, grass and shrub level (g/m^2^). The solid lines were the regression lines between plant production variables and elevation at significant differences at p < 0.05.

**Figure 5:**
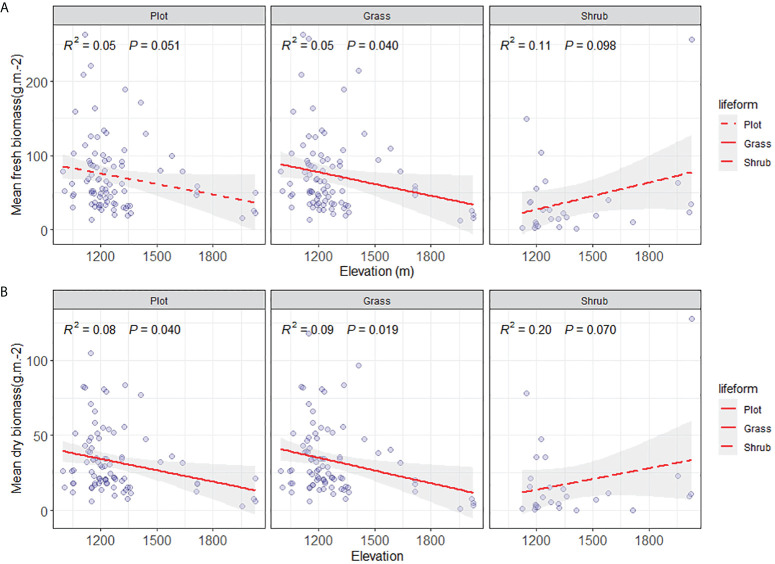
Comparison of aboveground biomass on community lifeform level. **(A)** Aboveground mean fresh biomass on plot, grass, and shrub level, and **(B)** Mean aboveground dry biomass on plot, grass, and shrub level (g/m^2^). The solid lines were the regression lines between plant production variables and elevation at significant differences at p < 0.05.

The valley-type Savanna vegetation below an altitude of 1700 m displays heterogeneous altitudinal gradient effects on the community’s fresh biomass, dry biomass, and litter biomass (**Figures 4**, **5**). At the community level, a distinct altitudinal gradient effect was not evident for fresh, dry, and litter biomass of the valley-type Savanna vegetation occurring below 1700 meters (*p* > 0.05). Upon segregating the shrub-herb component level, the increase in altitude does not prompt any significant alterations (*p* > 0.05) in the live and dry biomass of the herbaceous constituents within the vegetation. However, a discernible downward trend is observed in terms of litter biomass (*p* < 0.05). In contrast, a notable increasing trend (*p* > 0.05) is observed in the live and dry biomass of the shrub component within the vegetation, while the litter biomass remains relatively consistent (*p* > 0.05) (**Figure 4**). These findings collectively signify that the aboveground productivity of the valley-type Savanna vegetation exhibits a degree of stability at the cessation of the uncorrected stage. Nonetheless, the turnover of litter from herbaceous plants and the productivity of shrub plants are significantly influenced by altitude gradients. Concurrently, with the modulation of altitude gradients, the contribution of shrubs to the altitude gradient pattern of the dry and hot valley vegetation acquires an increasingly prominent role.

#### 3.2.3 Aboveground biomass distribution of plant functional groups in Yuanmou

Along the altitudinal gradient of 980 to 1700 meters in Yuanmou’s dry-hot valley, the functional groups showcase distinct biomass patterns. Specifically, in the community species functional group, the average fresh biomass of species exhibits a non-significant decreasing trend (*p* > 0.05), while the dry biomass demonstrates a significant reduction trend (*p* < 0.05). In the herbaceous functional group, both the average fresh and dry biomass exhibit a significant decrease (*p* < 0.05). The shrub functional group displays a non-significant increasing trend in both average fresh and dry biomass (*p* > 0.05) (**Figure 5**). This compellingly underscores the primary role of declining herbaceous species’ average biomass along the ascending altitudinal gradient as the key driver behind the decreased community productivity of the river valley Savanna vegetation. This observation significantly emphasizes the vital contribution of the herbaceous functional group in shaping the altitudinal gradient pattern of vegetation productivity within the distinctive context of the arid and hot river valley environment.

### 3.3 The environmental interpretation of elevation distribution patterns of valley-type Savanna

#### 3.3.1 Environmental effects on elevation distribution patterns of species diversity

**Figure 6:**
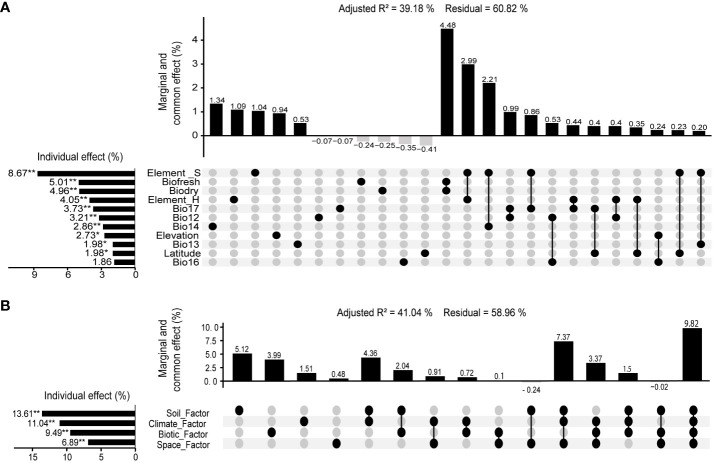
Variation partitioning analysis between community species diversity and environment in valley-type Savanna form Yuanmou dry-hot valley of Jinsha River basin, southwest China (n=111). **(A)** Environmental variables, and **(B)** Environmental factors.

The *RDA* results showed that including Elevation, Latitude, fresh biomass, dry biomass, BIO12 (Annual Precipitation, mm), BIO13 (Precipitation of Wettest Month, mm), BIO14 (Precipitation of Driest Month, mm), BIO16 (Precipitation of Wettest Quarter, mm), Bio17 (Precipitation of Driest Quarter, mm), Element_S, and Element_H, 11 environmental variables had an impact on community species diversity (Adjusted R^2 = ^0.555, p <0.01). The *VPA* results showed that environmental variables had a significant effect (*p* < 0.01) on community species diversity, with a total explanatory power of 39.18% (Monte Carlo permutation test for 999 times, *p* < 0.01). Among them, Element_S, above-ground fresh biomass, above-ground dry biomass, Element_H, Bio17, BIO12, BIO14, Elevation, BIO13, Latitude were the dominant environmental variables that affect community species diversity, with explanatory rates of 8.67% (*p* < 0.01), 5.01% (*p* < 0.01), 4.96% (*p* < 0.01), 4.05% (*p* < 0.05), 3.73% (*p* < 0.05), 3.21% (*p* < 0.05), 2.86% (*p* < 0.05), 2.73% (*p* < 0.05), 1.98% (*p* < 0.05) 1.98% (*p* < 0.05) respectively (**Figure 6A**).

The *RDA* and *VPA* results showed that spatial, vegetation, climate, and soil as four environmental factors have a significant effect (*p* < 0.01) on community species diversity, with a total explanatory power of 41.04% (Monte Carlo permutation test for 999 times, *p* < 0.01). Among them, the explanatory powers of environmental factors on community species diversities were as follows: soil factor (13.61%, *p* < 0.01) > climate factor (11.04%, *p* < 0.01) > vegetation factor (9.49%, *p* < 0.05) > spatial factor (6.89%, *p* < 0.01) (**Figure 6B**).

#### 3.3.2 Environmental effects on elevation distribution patterns of aboveground biomass

**Figure 7:**
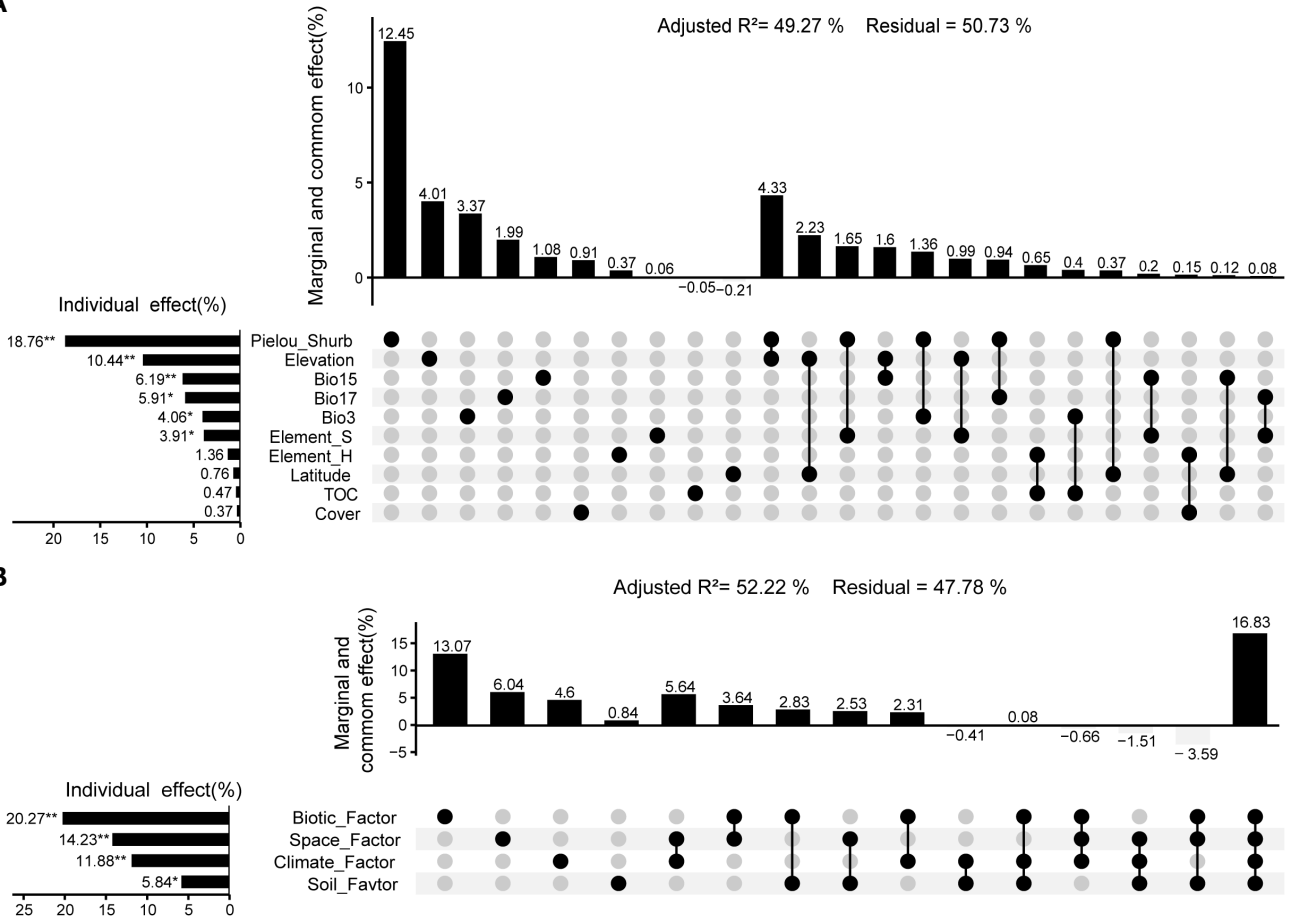
Variation partitioning analysis between aboveground biomass of community and environment in valley-type Savanna form Yuanmou dry-hot valley of Jinsha River basin, southwest China (n=111). **(A)** Environmental variables, and **(B)** Environmental factors.

The *RDA* results showed that 10 environmental variables, including Elevation, Longitude, Cover, Pielou-Shrub, Bio3 (Isothermality), Bio15 (Precipitation Seasonality (Coefficient of Variation)), Bio17 (Precipitation of Driest Quarter), TOC, Element_H, and Element_S, had a significant effect on the distribution of aboveground biomass (Adjusted R^2 = ^0.5551, *p* < 0.01). The *VPA* results showed that environmental variables have a significant effect (*p* < 0.05) on community aboveground biomass, with a total explanatory power of 49.27% (Monte Carlo permutation test *p* < 0.01). Among them, Pielou-Shrub, Elevation, Bio15 (Precipitation Seasonality (Coefficient of Variation)), Bio17 (Precipitation of Driest Quarter), Bio3 (Isothermality), Element_S were the dominant environmental variables that affect the aboveground biomass of the community, with explanatory powers of 18.76% (*p* < 0.01), 10.44% (*p* < 0.01), 6.19% (*p* < 0.01), 5.91% (*p* < 0.05), 4.06% (*p* < 0.05), and 3.91% (*p* < 0.05) respectively (**Figure 7A**).

The *RDA* and *VPA* results showed that four environmental factors spatial, vegetation, climate, and soil have an impact on the distribution of aboveground biomass. Variance decomposition analysis showed that environmental factors have a significant effect (*p* < 0.01) on community aboveground biomass, with a total explanatory power of 52.22% (Monte Carlo permutation test 999 times, *p* < 0.01). In this regard, the hierarchical order of explanatory power attributed to environmental factors on community biomass was as follows: vegetation-related factors (20.27%, *p* < 0.01) > spatial factors (14.23%, *p* < 0.01) > climatic factors (11.88%, *p* < 0.01) > soil-related factors (5.84%, *p* < 0.05) (**Figure 7B**).

## 4 Discussion

### 4.1 Elevation patterns of species richness in valley-type Savanna

The spatial configuration of plant species diversity along altitudinal gradients in mountainous environments represents a pervasive geographical phenomenon within terrestrial ecosystems, often expressed as vertical vegetation stratification (Gaston, 2000; Zheng et al., 2000). In this study, we unveil the botanical landscape of the dry-hot valley of Yuanmou, spanning altitudes below 2200 m above sea level, revealing five distinct vegetation assemblages along the altitudinal continuum: sparse tree Savanna, grass Savanna, shrub-grass Savanna, shrub-tree dwarf woodland, and montane coniferous-broadleaved mixed forest or evergreen broadleaved forest. This distribution profile elucidates a transitional character, traversing from arid to semi-arid conditions and further into temperate forests, thereby elucidating a discernible altitudinal gradient effect.

The riverine savanna biome predominates within elevations below 1700 meters. In contrast to the conventional vertical stratification of montane vegetation influenced by regional subtropical monsoon climates, the Jinsha River arid valley’s flora exhibits an inverted gradient. This phenomenon can primarily be attributed to the unique geographical and geological attributes of the valley, characterized by inverted habitat islands (Zhang, 1992; Shen, 2016). Additionally, the plant composition within this region leans towards a tropical and subtropical affiliation (Wu, 1965; Jin et al., 1987), substantiating prior investigations of arid valley vegetation within the transverse mountain ranges. This finding underscores our study’s efficacy in capturing the quintessence of riverine savanna vegetation, thereby providing a robust cornerstone for subsequent research determinations.

The modulation of species diversity along altitudinal gradients, encapsulating shifts in species richness and evenness, stands as a conspicuous feature characterizing vertical stratification in mountainous vegetation. Among the predominant patterns, two primary paradigms, namely linear relationships and unimodal curves, collectively account for fifty percent of antecedent research observations (Borcard et al., 2020). Intriguingly, the prevailing trend of monotonically increasing diversity along altitudinal gradients accounts for less than a quarter of documented instances (Rahbek, 2004). In our study, the riverine savanna biome predominantly flourishes within altitudes below 1700 meters, characterized by shrub and herbaceous dominance. As altitudes elevate, indices denoting species richness, Shannon-Wiener index, Simpson diversity index, and Pielou’s evenness index, collectively exhibit pronounced upward trajectories (*p* < 0.05), consistently evincing a monotonic escalation. This characteristic arises from the intricate interplay between environmental and biological determinants.

On one hand, the gradient shifts in temperature, water availability, and other ecophysiological variables, predominantly manifesting within the altitudinal gradient of the mountainous terrain, exert discernible influences on plant distributional dynamics (Gaston, 2000; Wang et al., 2021). The distinctive eco-geomorphic attributes of heightened temperature and aridity within the lower reaches of the arid valley, juxtaposed with diminished stress levels and amplified topographic variability at higher elevations, collectively engender a propitious milieu for the origination and perpetuation of species diversity. On the other hand, our investigations unveil distinct ecotonal zones around 1400 to 1600 m within the Yuanmou, culminating in augmented species diversity. Moreover, antecedent studies suggest intimately interwoven interspecific interactions amid savanna grassland species within the Yuanmou dry-hot valley (He et al., 2018), rendering them particularly responsive to anthropogenic disturbances, thereby precipitating discernible shifts in community compositional attributes (He et al., 2023). This underscores the role of coexistence mechanisms among species along altitudinal gradients in shaping the spatiotemporal distributional fabric of plant diversity. Nonetheless, our contemporary comprehension of these mechanisms within the context of arid valley savanna vegetation remains embryonic, thus beckoning further empirical elucidation.

### 4.2 Elevational aboveground biomass patterns of valley-type Savanna

This study elucidates distinct alterations in both aboveground biomass and litter biomass across altitudinal gradients within the Yuanmou dry-hot valley savanna ecosystem (*p* < 0.05). Notably, there exists a conspicuous attenuation in biomass with increasing elevation, primarily attributed to the diminishing aboveground biomass driven by the reduction in herbaceous plant biomass. Presently, empirical investigations into the altitudinal gradient patterns of vegetation biomass in the arid valley remain relatively scant. Given the homologous nature of altitudinal and latitudinal gradients, often serving as scaled-down models (Körner, 2000), insights from both paradigms are synthesized to unravel the salient attributes of the Jinsha River arid valley savanna vegetation.

Across latitudinal gradients, savanna ecosystems within drainage basins, such as Yuanjiang, Dadu River, and Minjiang River, generally manifest an ascending trend in both community and shrub biomass with increasing latitude. However, the dynamism of herbaceous biomass remains less transparent, whereas litter biomass exhibits a marked reduction (Wang et al., 2022). This incongruity underscores the pivotal role of shrubs in shaping the biomass distribution pattern of the arid valley vegetation, divergent from the prevailing deduction of herbaceous biomass dominance drawn from our study. The potential contributory factors to this divergence encompass scale disparities between altitudinal and latitudinal gradients (Richards et al., 1975), disparities in environmental attributes entwined with intricate terrain (Zhang et al., 2019), alongside conceivable sampling errors or biases (Borcard et al., 2020).

Based on antecedent on-site observations, the arid valleys of southwestern China can be dichotomized into two topographical archetypes: basin-type valleys, characterized by substantial incisions and relatively expansive alluvial plains at their base, typified by the Yuanmou Basin; and gorge-type valleys, distinguished by pronounced river incision with precipitous valley walls, pervading much of the arid valleys within the transverse mountain ranges. Basin-type valleys exhibit gradual topographical transitions, often presenting a mosaic of shrubland-grassland-tree communities. In contrast, gorge-type valleys, owing to their substantial elevation differentials and dynamic terrain, house shallow soils or exposed bedrock, fostering the persistence of resilient shrub communities while demonstrating a paucity of distinct grassland or shrub-grassland mosaic communities. Acknowledging these realities, we posit that prevailing large-scale research mandates a deeper exploration of the altitudinal distribution patterns of herbaceous plants within arid valley savanna vegetation. Similarly, facets influencing productivity, community composition, and the architecture of species assemblages warrant a comprehensive reassessment (Zhang et al., 2019).

Furthermore, it is imperative to underscore that both altitudinal (as discerned in Section 3.2 of this study) and latitudinal (as documented by Wang (Wang et al., 2022)) biomass patterns within the arid valley consistently evince a decremented trend in litter biomass with ascending altitude or latitude. This is significantly different from the pattern of an overall decrease in aboveground biomass of herbaceous swamp vegetation on the Qinghai Tibet Plateau with increasing altitude (Shen et al., 2021), and consistent with the pattern of an Evergreen Andean–Amazonian Forest in Ecuador (Maza et al., 2022). Along the altitudinal gradient, the nadir of litter biomass within the riverine savanna community is concentrated within the elevation range of 1500-1700 meters, emblematic of the upper demarcation of this valley’s savanna vegetation (as expounded upon in Section 3.1 of this study). Remarkably, this tract concurrently serves as a prolific genesis of intense soil erosion on slopes, corroborated by preceding field observations. These collective findings accentuate the upper boundary of the arid valley savanna vegetation as the most receptive domain concerning the intricate interplay between valley vegetation and environmental flux. Consequently, it should assume a pivotal prominence in subsequent research endeavors addressing ecological rejuvenation, soil preservation, and broader eco-environmental imperatives within the region.

### 4.3 Effects of environmental factors on the elevation distributions of plant diversity and ANPP

Since Humboldt’s pioneering investigations into altitudinal variations in tropical American vegetation, the exploration of the rules governing the formation and distribution dynamics of biodiversity in mountainous environments has persisted unceasingly. The prevailing view that mountainous terrains constitute biodiversity hotspots has gained broad recognition (Rahbek et al., 2019). The vegetation of the arid valleys in southwestern China, designated as valley-type savanna vegetation, represents a distinct scenario shaped by the topographical isolation arising from the uplift of the Qinghai-Tibet Plateau. This isolation has engendered the establishment of unique inverted habitat islands, where towering mountain ridges segregate analogous habitats, resulting in relatively enclosed landscapes within the transverse mountain ranges (Shen, 2016). Consequently, the biodiversity in this region exemplifies semi-arid to arid vegetation and flora, characterized by cryptic and relict traits under the influence of the tropical and subtropical humid monsoon climate (Wu, 1965; Zhang, 1992). Crucial environmental factors encompassing moisture, temperature, soil, and topography exert pivotal control over species diversity and aboveground biomass (Hawkins et al., 2003). This underscores that the species diversity and community productivity of the valley-type Savanna vegetation could be substantially impacted by spatial localization, climatic conditions, soil attributes, and intrinsic vegetation adaptations.

This current study unravels the substantial influences of four environmental factors – spatial, vegetation, climate, and soil – on species diversity and aboveground biomass within the valley savanna vegetation. Spatial localization emerges as a determining factor governing plant species diversity and productivity, directly or indirectly condensing environmental variables such as temperature and moisture, alongside biological facets such as vegetation type and species composition (Gaston, 2000; Wang et al., 2021). Notably, spatial factors wield marked influences on both species diversity (**Figure 5**) and aboveground biomass (**Figure 6**) of the valley savanna vegetation, with elevation assuming a pivotal role in reflecting variations in species diversity and aboveground biomass. The southeastern mountain ranges of the Qinghai-Tibet Plateau recurrently intercept and precipitate substantial monsoonal moisture, inducing pronounced water stress within certain deep valley vegetation, which concurrently manifests an atypical descent towards both mountain ridges and valleys (Zhang et al., 2019). This deduction lucidly expounds on the impact of bioclimatic factors, primarily revolving around moisture and energy variables, on the altitudinal gradient patterns of species diversity and aboveground biomass within the Yuanmou arid valley savanna.

Furthermore, the distinctive environmental conditions of the arid valley profoundly shape the intricate interplay between regional vegetation and soil. This dynamic interaction confers parallel and heightened explanatory power to both vegetation and soil factors concerning the altitudinal gradient patterns of species diversity and aboveground biomass (**Figures 5**, **6**). Remarkably, this study underscores that species diversity patterns are primarily orchestrated by soil and bioclimatic factors, whereas the distribution of aboveground biomass is predominantly driven by vegetation and spatial factors (**Figures 5**, **6**). This implies that the altitudinal gradient patterns of species diversity and aboveground biomass within the valley savanna vegetation evince divergent modes of response to the environmental factors and variables of the arid valley. Furthermore, within this study, variables comprising vegetation-environmental factors, such as species diversity indices (particularly shrub evenness, Pielou_Shurb, **Figure 4**, 18.76%), constitute the most substantial (20.27%) portion of explanatory prowess regarding the altitudinal gradient pattern of aboveground biomass. Conversely, variables encompassing vegetation-environmental factors, including community standing biomass (4.96%) and fresh biomass (5.01%), contribute relatively less (9.49%) to elucidating plant species diversity. This underscores that the mutual relationship between species diversity and aboveground biomass does not exhibit commensurate contributions to their respective altitudinal gradient patterns. Thus, these findings support the hypothesis positing biodiversity as the bedrock of vegetation productivity (Reich et al., 1997). Nonetheless, congruent with the work of Grace et al. (2016), this study partially challenges the notion that vegetation productivity propels the formation of species diversity.

## 5 Conclusion

The species distribution below 2200 meters in the study area exhibits a typical altitudinal gradient effect, which is commonly observed in ecological studies. In the valley of the study area, the vegetation composition primarily consists of tropical sparse grassland, with a prevalence of shrubs and herbaceous plants, occurring mainly below 1700 meters. Diversity indices including the species richness index of *SR* and *Shannon-Wiener*, and the species evenness index of *Simpson* and *Pielou* were significantly increased (*p* < 0.05) along the altitude gradient. The aboveground biomass productivity of the community shows a decrease at higher altitudes (*p* < 0.05), with the reduction in herbaceous biomass being the major contributing factor to this decline. Environmental factors and variables play a noticeable role in shaping the distribution pattern of species and community biomass. Nevertheless, the patterns of species diversity and aboveground biomass along the altitudinal gradient in the valley-type Savanna vegetation reveal distinctive responses to the arid and hot environmental conditions in the valley. Furthermore, the relationship between species diversity and aboveground biomass, in terms of their altitudinal gradient patterns, does not exhibit equal trends. Soil and climatic environmental factors and their variables explained 60.06% of the altitudinal gradient pattern of species diversity in valley-type Savanna, while the regulatory role of plant species or functional groups alone explained 38.82% of the altitudinal gradient pattern of community biomass. Thus, this study demonstrates that the spatial pattern of valley-type Savanna is shaped by differences in environmental responses to climate-soil factors that screen plant species or functional groups and the productive capacity of the retained species, and highlights the suitability of Yuanmou’s arid and hot valley as a microcosm experimental field for comprehensively investigating the intricate relationship between savanna vegetation and environmental factors.

## Data availability statement

The raw data supporting the conclusions of this article will be made available by the authors, without undue reservation.

## Author contributions

HG: Data curation, Formal analysis, Funding acquisition, Investigation, Visualization, Writing – original draft, Writing – review & editing, Methodology, Software, Validation. SZ: Conceptualization, Data curation, Funding acquisition, Methodology, Supervision, Visualization, Writing – original draft. FH: Data curation, Formal analysis, Methodology, Resources, Validation, Visualization, Writing – original draft. SL: Data curation, Formal analysis, Investigation, Writing – review & editing. WY: Data curation, Formal analysis, Investigation, Writing – review & editing. YH: Data curation, Formal analysis, Investigation, Writing – review & editing. YB: Data curation, Formal analysis, Visualization, Writing – original draft. YC: Formal analysis, Investigation, Resources, Writing – review & editing. JY: Formal analysis, Investigation, Writing – review & editing. LQ: Formal analysis, Investigation, Writing – review & editing. LZ: Formal analysis, Investigation, Writing – review & editing. QJ: Formal analysis, Investigation, Writing – review & editing.

## References

[B1] Albrich K. Rammer W. Seidl R. (2020). Climate change causes critical transitions and irreversible alterations of mountain forests. Global Change Biol. 26, 4013–4027. doi: 10.1111/gcb.15118 PMC7317840 32301569

[B2] Bai Y. Han X. Wu J. Chen Z. Li L. (2004). Ecosystem stability and compensatory effect in the Inner Mongolia grassland. Nature. 431, 181–184. doi: 10.1038/nature02850 15356630

[B3] Beniston M. (2003). Climatic change in mountain regions: A review of possible impacts. Climatic Change. 59, 5–31. doi: 10.1023/A:1024458411589

[B4] Borcard D. Gillet F. Legendre P. (2020). Numerical ecology with R, 2nd Chinese edition. Ed. Lai J. (Beijing: Institute of Botany, Chinese Academy of Sciences Higher Education Press).

[B5] Cuesta F. Muriel P. Llambí L. D. Halloy S. Aguirre N. Beck S.. (2017). Latitudinal and altitudinal patterns of plant community diversity on mountain summits across the tropical Andes. Ecography. 40, 1381–1394. doi: 10.1111/ecog.02567

[B6] Fang J. Song Y. Liu H. Pu S. (2002). Vegetation climate relationship and its application in the division of vegetation zone in China. Acta Botanica Sin., 1105–1122.

[B7] Fang J. Wang X. Shen Z. Tang Z. He J. Yu D.. (2009). Methods and protocols for plant community inventory. Biodiversity Science. 17, 533–548. doi: 10.3724/SP.J.1003.2009.09253

[B8] Feldmeier S. Schmidt B. R. Zimmermann N. E. Veith M. Ficetola G. F. Lötters S. (2020). Shifting aspect or elevation? The climate change response of ectotherms in a complex mountain topography. Diversity Distributions. 26, 1483–1495. doi: 10.1111/ddi.13146

[B9] Fick S. E. Hijmans R. J. (2017). WorldClim 2: new 1-km spatial resolution climate surfaces for global land areas. Int. J. climatology. 37, 4302–4315.

[B10] Gaston K. J. (2000). Global patterns in biodiversity. Nature. 405, 220–227. doi: 10.1038/35012228 10821282

[B11] Grace J. Anderson T. M. Seabloom E. W. Borer E. T. Adler P. B. Harpole W. S.. (2016). Integrative modeling reveals mechanisms linking productivity and plant species richness. Nature. 529, 390–393. doi: 10.1038/nature16524 26760203

[B12] Guo M. Gao L. Fan C. (2019). Research progress on species diversity and productivity. World Forestry Ｒesearch. 32, 03, 18–23. doi: 10.13348/j.cnki.sjlyyj.2019.0007.y

[B13] Hawkins B. A. Field R. Cornell H. V. Currie D. J. Guégan J.-F. Kaufman D. M.. (2003). Energy, water, and broad-scale geographic patterns of species richness. Ecology. 84, 3105–3117. doi: 10.1890/03-8006

[B14] He Y. Chen W. (1997). A review of gradient changes in species diversity of land plant communities. Acta Ecologica Sinica. 1997 (01), 93–101. doi: 10.3321/j.issn:1000-0933.1997.01.014

[B15] He Y. Huang C. (1995). Soil taxonomic classification in Yuanmou dry and hot valley, Yunnan province. Mountain Res. 13, 73–78. doi: 10.16089/j.cnki.1008-2786.1995.02.002

[B16] He G. Shi Z. Yan B. Yang H. Sun Y. Wang Y.. (2023). Effects of fencing enclosure on interspecific associations in a Savanna grassland community in China's arid-hot valley region. Acta Prataculturae Sinica. 32, 1–14. doi: 10.11686/cyxb2022080

[B17] He G. Yan B. Ji Z. Shi L. Fan B. Fang J.. (2018). Interspecific relationship in Savanna grassland in Yuanmou, a dry-hot valley upstream of the Yangtze River. Acta Prataculturae Sinica. 27, 62–71. doi: 10.11686/cyxb2017300

[B18] Hodkinson I. D. (2005). Terrestrial insects along elevation gradients: species and community responses to altitude. Biol. Rev. 80, 489–513. doi: 10.1017/S1464793105006767 16094810

[B19] Hua T. Zhao W. Cherubini F. Hu X. Pereira P. (2021). Sensitivity and future exposure of ecosystem services to climate change on the Tibetan Plateau of China. Landscape Ecology. 36, 3451–3471. doi: 10.1007/s10980-021-01320-9 34456507 PMC8382670

[B20] Jiang H. (1980). Distributional features and zonal regularity of vegetation in Yunnan. Acta Botanica Yunnanica, 22–32.

[B21] Jin Z. Ou X. Zhou Y. (1987). The general situation of natural vegetation in dry-hot river valley of Yuanmou, Yunnan province. Acta phytoecologica geobotanica sinica. 11, 308–317.

[B22] Jobbágy E. G. Paruelo J. M. León R. J. C. (1996). Vegetation heterogeneity and diversity in flat and mountain landscapes of Patagonia-(Argentina). J. Vegetation Sci. 7, 599–608.

[B23] Körner C. (2000). Why are there global gradients in species richness? Mountains might hold the answer. Trends Ecol. Evolution. 15, 513–514. doi: 10.1016/S0169-5347(00)02004-8

[B24] Kou Z. Yao Y. Hu Y. Zhang B. (2020). Discussion on position of China’s north-south transitional zone by comparative analysis of mountain altitudinal belts. J. Mountain Science. 17, 1901–1915. doi: 10.1007/s11629-019-5893-x

[B25] Lai J. Zou Y. Zhang J. Peres-Neto P. R. (2022). Generalizing hierarchical and variation partitioning in multiple regression and canonical analyses using the rdacca.hp R package. Methods Ecol. Evolution. 13, 782–788. doi: 10.1111/2041-210x.13800

[B26] Liang J. Ding Z. Lie G. W. Zhou Z. Singh P. B. Zhang Z.. (2020). Species richness patterns of vascular plants and their drivers along an elevational gradient in the central Himalayas. Global Ecology and Conservation 24, e01279. doi: 10.1016/j.gecco.2020.e01279

[B27] Liang H. Liu L. Fu T. Gao H. Li M. Liu J. (2022). Vertical distribution of vegetation in mountain regions: A review based on bibliometrics. Chin. J. Eco-Agriculture 30, 1077–1090. doi: 10.12357/cjea.20210858

[B28] Liu Y. (2015). Plant diversity and phytogeography of arid valley in major rivers of the south western China (Beijing: Peking University). PhD dissertation.

[B29] Liu B. (2021). Recent advances in altitudinal distribution patterns of biodiversity. Ecol. Environ. Sci. 30, 438–444. doi: 10.16258/j.cnki.1674-5906.2021.02.0025

[B30] Liu G. Ji Z. Fang H. Yang Y. Sha Y. (2011). Ecological response and evaluation of typical recovery mode for dry-hot valley degenerate ecosystem (Beijing: Press of Science).

[B31] Liu X. Shi Z. Yang D. Liu S. Yang Y. Ma Q. (2005). Advances in study on changes of biodiversity and productivity along elevational gradient in mountainous plant community. World Forestry Res. 04), 27–34. doi: 10.13348/j.cnki.sjlyyj.2005.04.006

[B32] Liu Y. Yu X. Yu Y. Hu W.-H. Lai J.-S. (2023). Application of “rdacca.hp” R package in ecological data analysis: case and progress. Chin. J. Plant Ecology. 47, 134–144. doi: 10.17521/cjpe.2022.0314

[B33] Liu Y. Zhu X. Shen Z. Sun H. (2016). Flora compositions and spatial differentiations of vegetation in dry valleys of Southwest China. Biodiversity Science. 24, 367–377. doi: 10.17520/biods.2015240

[B34] Loreau M. Naeem S. Inchausti P. Bengtsson J. Grime J. P. Hector A.. (2001). Biodiversity and ecosystem functioning: current knowledge and future challenges. Science. 294, 804–808. doi: 10.1126/science.1064088 11679658

[B35] Maza B. Rodes-Blanco M. Rojas E. (2022). Aboveground biomass along an elevation gradient in an evergreen andean–amazonian forest in Ecuador. Front. Forests Global Change. 5. doi: 10.3389/ffgc.2022.738585

[B36] Niu K. Chu C. Wang Z. (2022). Dynamic niche: a new foundation for rebuilding theory of community ecology. Scientia Sin. Vitae 52, 403–417. doi: 10.1360/SSV-2021-0160

[B37] Niu K. Liu Y. Shen Z. He F. Fang J. (2009). Community assembly: the relative importance of neutral theory and niche theory. Biodiversity science 17, 06, 579–593. doi: 10.3724/SP.J.1003.2009.09142

[B38] Oksanen J. Simpson G. Blanchet F. Kindt R. Legendre P. Minchin P.. (2022). Vegan: community ecology package_ (R package version 2.6-2). Available at: https://CRAN.R-project.org/package=vegan.

[B39] Ou X. (1988). The study of flora in Yuanmou dry-hot river valley. Acta Botanica Yunnanica. 10, 11–18.

[B40] Qu Z. (1957). Some basic concepts of Yunnan vegetation. J. Yunnan University(Natural Sci. Edition), 45–53.

[B41] Rahbek C. (2004). The role of spatial scale and the perception of large-scale species-richness patterns. Ecol. Letters. 8, 224–239. doi: 10.1111/j.1461-0248.2004.00701.x

[B42] Rahbek C. Borregaard M. K. Colwell R. K. Dalsgaard B. Holt B. G. Morueta-Holme N.. (2019). Humboldt's enigma: What causes global patterns of mountain biodiversity? Science. 365, 1108–1113. doi: 10.1126/science.aax0149 31515383

[B43] Rangwala I. Miller J. R. (2012). Climate change in mountains: a review of elevation-dependent warming and its possible causes. Climatic Change. 114, 527–547. doi: 10.1007/s10584-012-0419-3

[B44] R Core Team (2022). R: A language and environment for statistical computing (Vienna, Austria: R Foundation for Statistical Computing). Available at: https://www.R-project.org/.

[B45] Reich P. B. Walters M. B. Ellsworth D. S. (1997). From tropics to tundra: global convergence in plant functioning. Proc. Natl. Acad. Sci. United States America. 94 25, 13730–13734. doi: 10.1073/PNAS.94.25.13730 PMC28374 9391094

[B46] Richards P. W. Walter H. Wieser J. D. (1975). Vegetation of the earth in relation to climate and the eco-physiological conditions. J. Ecology. 63, 1014. doi: 10.2307/2258632

[B47] Shen Z. (2016). Plant diversity in the dry valleys of Southwest China: spatial deviation and determinants for flora and plant communities. Biodiversity Science. 24, 363–366. doi: 10.17520/biods.2016049

[B48] Shen X. Jiang M. Lu X. Liu X. Liu B. Zhang J.. (2021). Aboveground biomass and its spatial distribution pattern of herbaceous marsh vegetation in China. Sci. China Earth Sci. 51, 1306–1316. doi: 10.1007/s11430-020-9778-7

[B49] Tilman D. (2000). Causes, consequences and ethics of biodiversity. Nature. 405, 208–211. doi: 10.1038/35012217 10821280

[B50] Tilman D. Wedin D. A. Knops J. M. H. (1996). Productivity and sustainability influenced by biodiversity in grassland ecosystems. Nature. 379, 718–720. doi: 10.1038/379718A0

[B51] Wang Z. Hu B. Bao W. Li F. Hu H. Wei D.. (2022). Latitudinal patterns and underlying factors of component biomass in plant communities in the arid valley of southwest China. Chin. J. Plant Ecology. 46, 539–551. doi: 10.17521/cjpe.2021.0237

[B52] Wang S. Luo M. Feng Y. Chu C. Zhang D. (2022). Theoretical advances in biodiversity research. Biodiversity Sci. 30, 25–37. doi: 10.17520/biods.2022410

[B53] Wang J. Yu C.-q. Fu G. (2021). Warming reconstructs the elevation distributions of aboveground net primary production, plant species and phylogenetic diversity in alpine grasslands. Ecological Indicators 133, 108355. doi: 10.1016/j.ecolind.2021.108355

[B54] Wickham H. (2016). ggplot2: elegant graphics for data analysis Vol. 2016 (Springer-Verlag New York) Berlin: Springer International Publishing.

[B55] Wu Z. (1965). Tropical kinship of chinese flora. Chinese Science Bulletin, 1, 25–33.

[B56] Wu Z. (1979). On the zoning of Chinese flora. Acta botanica Yunnanica, 1, 1–20.

[B57] Yan B. Ji Z. Fan B. Wang X. He G. Shi L.. (2016). Plants adapted to nutrient limitation allocate less biomass into stems in an arid-hot grassland. New Phytologist. 211, 1232–1240. doi: 10.1111/nph.13970 27101947

[B58] Yang Y. Han J. Liu Y. Zhongyong C. Shi S. Sina C.. (2016). A comparison of the altitudinal patterns in plant species diversity within the dry valleys of the Three Parallel Rivers region, northwestern Yunnan. Biodiversity Science. 24, 440–452. doi: 10.17520/biods.2015361

[B59] Yang D. Wang Y. S. D. Wang Q. Ke Y. Zhang Y. B. Zhang S. B.. (2023). Physiological response and photosynthetic recovery to an extreme drought: Evidence from plants in a dry-hot valley savanna of Southwest China. Sci. Total Environ., 161711. doi: 10.1016/j.scitotenv.2023.161711 36682563

[B60] Zhang R. (1992). The dry valley of the Henduan Mountains region (Beijing: Science Press).

[B61] Zhang W. Liu L. Song K. Li X. Wang Y. Tang Y.. (2019). Remote sensing the orographic effects of dry-hot valley on vegetation distribution in the southeast Tibetan Plateau. Int. J. Remote Sensing. 40, 8589–8607. doi: 10.1080/01431161.2019.1620370

[B62] Zhang J.-L. Poorter L. Cao K.-f. (2012). Productive leaf functional traits of Chinese savanna species. Plant Ecol. 213, 1449–1460. doi: 10.1007/s11258-012-0103-8

[B63] Zhang J. Wang D. Wang Y. Wen A. (2000). Discusses on eco-environnment changes in dry-hot valley of Yuanmou. Scientia geographica sinica. 20, 148–152. doi: 10.3969/j.issn.1000-0690.2000.02.010

[B64] Zheng D. Zhang Q. Wu S. (2000). Mountain geoecology and sustainable development of the Tibetan Plateau (Berlin: Springer (Springer Science+Business Media B.V). doi: 10.1007/978-94-010-0965-2

